# 

**Published:** 1897-04

**Authors:** 


### MARRIAGES.

On January 30th Dr. Wilfred Lellman, Professor at the New York College of Veterinary Surgeons, N. Y., to Miss Gertrude Runge, of Stettin, Germany.

At Detroit, Mich., November 18, 1896, by the Rev. C. H. Vincent, Dr. Alexander Findlay, of Camden, N. Y., to Miss Lillian McAully, of Centerville, Ont., Canada.

### PERSONAL.

Dr. John W. Adams was a participant in the annual reunion and dinner of the Philadelphia Chapter of the Psi Upsilon fraternity.

Mr. J. H. Brigham, Master of the National Grange, has been tendered and will accept the post of Assistant Secretary of Agriculture.

Veterinarian A. T. Sellers, of Camden, New Jersey, takes an active interest in the work of the Humane Society of that city.

Dr. A. K. Robertson, of Brooklyn, N. Y., has been on the sick-list. We are glad to report his convalescence, and trust that it may be complete.

Dr. Charles Bland, of Philadelphia, has been a sufferer from a severe sprain of the arm received in the performance of his professional duties.

Dr. James Fairley is associated with his brother in the ownership of a Broad Street livery stable.

Dr. William J. Denton, of Philadelphia, is nursing an injured arm, the result of a bite of one of his canine patients.

Dr. J. L. Ronan, of Corning, New York, after an illness which confined him to his bed for six months, is again at his post of duty.

Veterinarian W. J. Waugh, of the United States Army, has come east on a fifteen days' leave of absence. He will spend a good portion of his time with his brother, James A. Waugh, of Pittsburg.

Dr. W. B. E. Miller, of Camden, N. J., will be a candidate for reinstatement to a position in the Bureau of Animal Industry under the new administration.

Veterinarian W. N. Leavy, of New York City, has placed on the streets a very attractive canine ambulance in connection with his hospital.

Dr. George H. Bailey, of Deering, addressed the Maine Academy of Medicine and Science on February 8th on "Animal Tuberculosis." Those assigned as speakers in the discussion were Drs. J. F. Hill, Jesse A. Randall, Enoch Adams, Ernest W. Russell, A. L. Stanwood, Ex-Governor Cleaves, and Colonel Dow.

Veterinarian Hugh F. Doris, a student of law at a West Virginia college, has become the defendant in a $20,000 law-suit, the outgrowth of a horse deal.

The Western Humane Society of Pittsburg, Pa., has made the following appointments of veterinarians for the present year: Dr. James A. Waugh for Pittsburg proper, Dr. N. Rectenwald for the south side, Dr. H. S. Richards for the east end, and Dr. Charles M. Bond for the Allegheny district. This adoption of a staff of veterinarians will increase the Society's usefulness and facilitate prompt action whenever required.

Dr. E. H. Flood, of Philadelphia, late inspector of cattle for foreign shipment, has formed a copartnership with Dr. John A. Pearson, of Chicago. They have purchased the practice and hospital of the late Dr. William Tag.

At the twenty-fifth annual meeting of the Wisconsin Dairymen's Association in February Prof. E. A. A. Grange gave a practical address on diseases of the udder in cattle.

The Doylestown, Pa., *Intelligencer* of February 5th contains a very pointed article on bovine tuberculosis, for cattle-owners and dairymen, from the pen of President Ridge, of the State Association.

Veterinarian C. Howard Davidson, of Millbrook, New York, recently became a member of the American Berkshire Association.

Veterinarian J. D. O'Rourke, of San Francisco, Cal., holds the position of veterinarian to the city and county boards of health of that district.

Dr. John W. Adams has an excellent article on the bar-shoe in the *Horseshoers' Journal* for February and March.

Dr. E. H. Sheppard, of Cleveland, Ohio, is lecturing to the shoers of that city. Dr. M. J. Dunn, at Detroit, and Dr. Carter, at Saginaw, Michigan, are performing the same services for the shoers.

The United States Veterinary College at Washington has instituted a course of lectures for the horseshoers of the capital city.

Dr. F. C. Grenside, formerly of Guelph, Canada, has engaged in the export trade of horses to Europe. With others he has formed the American Horse Export Company.

Dr. George H. Bailey, of Portland, Maine, has been elected a Vice-President of the International Horsemen's Association recently organized at Chicago, Ill.

Veterinarian W. B. Stauffer, of Philadelphia, has opened an equine and canine infirmary in connection with his outdoor practice.

Dr. Charles Gresswell has just been reappointed State Veterinarian for Colorado by Governor Adams for a term of two years. This is a third term for Dr. Gresswell, he having been appointed by three different Governors.

Veterinarians F. S. Roop and George C. Faville hold the position of Assistant State Veterinarians in Virginia.

Dr. F. S. Shannon and H. B. Adair, recently stationed at Milwaukee in the meat-inspection department, have been transferred to the quarantine division, and assigned to duty at ports of entry on the Mexican border.

Ex-Secretary H. P. Rogers, of the Massachusetts Veterinary Association, was a visitor to the JOURNAL office in March. His vacation of a few days enabled him to visit Washington also.

Dr. James A. Waugh was veterinarian to the Duquesne Kennel Club show in March.

Secretary Edge, of the Department of Agriculture of Pennsylvania, has been compelled by illness to cease work, and on a leave of absence has gone on a trip in search of rest and renewed health. We wish for him an early and complete recovery.

Veterinary Surgeon Desmond, of Warrnambool, Victoria, has been appointed teacher of bacteriology and microscopical technology at the Melbourne Veterinary College.

Dr. A. W. Knight Tuck, of Melbourne, Australia, succeeds to the practice of Veterinary Surgeon Desmond at Warrnambool, Victoria.

Professor R. S. Huidekoper, of New York City, has recently received an appointment from the government of South Africa as resident inspector at the port of New York for all live-stock destined for shipment to any portion of the above-named country.

Dr. James A. Waugh, of Pittsburg, has purchased the practice and leased the hospital of Dr. John Doris, deceased.

Dr. David Waugh, graduate of the Indianapolis Veterinary College, will take an assistantship with his brother at Pittsburg.

Dr. R. S. Huidekoper, of the JOURNAL, will deliver in March and April a course of four lectures at the American Horse Exchange, New York City, on the exterior of the horse.

W. J. Hinman, V.S., of Winnipeg, Manitoba, has just returned from a trip to England.

Dr. J. C. McNeil and Superintendent of Police Lesley, of Pittsburg, were visitors to the JOURNAL office in March.

Dr. G. A. Johnson has been reappointed city meat-inspector for Sioux City, Iowa.

State Veterinarian Pearson has become a charter member of the Nittany Rod and Gun Club, with special rights and privileges in Centre County, Pennsylvania.

The experience of Dr. D. D. Lee, of Boston, in castrating the wrong donkey, terminating in a law-suit for damages, with a verdict for the plaintiff, adds another experience in the line of responsibilities that continue to heap themselves on the shoulders of the profession. Two donkeys on opposite corner lots seem loaded with danger to the approaching veterinarian.

